# A potential mechanism of the onset of acute eosinophilic pneumonia triggered by an anti‐PD‐1 immune checkpoint antibody in a lung cancer patient

**DOI:** 10.1002/iid3.238

**Published:** 2018-11-21

**Authors:** Takayuki Jodai, Chieko Yoshida, Ryo Sato, Yosuke Kakiuchi, Nahoko Sato, Shinji Iyama, Tomoko Kimura, Koichi Saruwatari, Sho Saeki, Hidenori Ichiyasu, Kazuhiko Fujii, Yusuke Tomita

**Affiliations:** ^1^ Department of Respiratory Medicine Graduate School of Medical Sciences Kumamoto University Kumamoto‐shi Kumamoto Japan

**Keywords:** Acute eosinophilic pneumonia, immune checkpoint blockade, immune‐related adverse event, lung cancer, programed cell death‐ligand 2 (PD‐L2)

## Abstract

**Introduction:**

The impact of immune checkpoint blockade on immunity in cancer patients is not completely elucidated due to the complexity of the immune network. Recent studies have revealed a significant role of programed cell death‐ligand 2 (PD‐L2) in negatively controlling the production of CD4+ T helper type 2 (Th2) cytokines and airway hypersensitiveness, suggesting hypo‐responsive Th2 cells via the PD‐1/PD‐L2 inhibitory pathway in lung could be reawaken by PD‐1 blockade therapy.

**Methods:**

We describe the first report of acute eosinophilic pneumonia (AEP), which is known as Th2‐associated pulmonary disease, triggered by nivolumab, an anti‐PD‐1 antibody, in an advanced non‐small cell lung cancer patient. Based on the current case report and literature, the present study proposes a potential mechanism of the onset of AEP as an immune‐related adverse event (irAE).

**Results:**

A 62‐year‐old man was diagnosed with lung adenocarcinoma and nivolumab was selected as the third‐line regimen. After three cycles of nivolumab treatment, chest computed tomography revealed pulmonary infiltrates in both lungs. The patient was diagnosed with AEP based on the diagnostic criteria for AEP. Nivolumab was suspended and the patient was started on oral prednisolone. His symptoms and radiological findings had rapidly improved.

**Conclusions:**

Given the increasing frequency of the use of anti‐PD‐1 antibodies, clinicians should be aware of the risk of AEP as a potential irAE. This study may improve our understanding of the pathophysiology underlying Th2‐associated irAEs and AEP.

## 1 INTRODUCTION

The impact of immune checkpoint blockade on immunity in cancer patients is not completely elucidated due to the complexity of the immune network. Recent studies have revealed a significant role of programed cell death‐ligand 2 (PD‐L2) in negatively controlling the production of CD4+ T helper type 2 (Th2) cytokines and airway hypersensitiveness,^1^, ^2^, ^3^ suggesting hypo‐responsive T helper 2 cells (Th2 cells) via the programed cell death‐1 (PD‐1)/programed cell death‐ligand 2 (PD‐L2) inhibitory pathway in lung could be reawaken by PD‐1 blockade therapy.

Acute eosinophilic pneumonia (AEP) is a Th2 inflammation associated lung disease with a remarkable increase in bronchoalveolar lavage (BAL) eosinophils, first described in 1989.^4^, ^5^ Although AEP has been associated with tobacco smoke, environmental or occupational dust exposures, toxin inhalations, and medications including NSAIDs, minocycline, cephalosporins, and phenytoin,^5^, ^6^, ^7^ nivolumab‐induced AEP has not been reported. In addition, the precise mechanism of significant eosinophil accumulation in AEP remains to be elucidated.

Here we describe the first report of AEP triggered by nivolumab, an anti‐PD‐1 antibody, in an advanced non‐small cell lung cancer patient. Based on the current case, present study proposes a potential mechanism of the onset of AEP as an immune‐related adverse event (irAE).

## 2 CASE PRESENTATION

**Figure 1:**
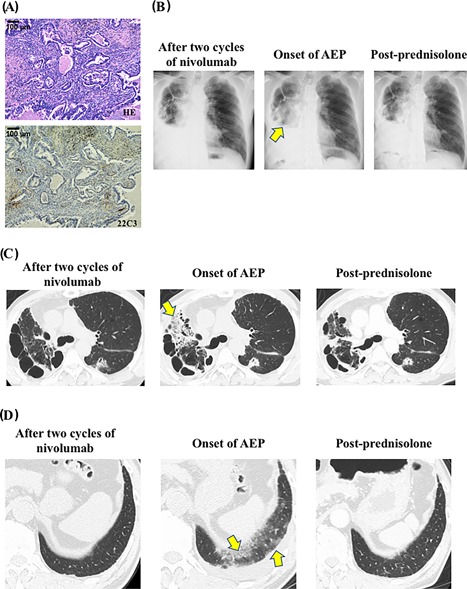
Key pathology and imaging (A) A hematoxylin and eosin staining and an immunohistochemical staining of primary lung tumor negative staining for PD‐L1 (clone 22C3 pharmDx kit, tumor proportion score <1%). B, Chest radiograph after two cycles of nivolumab (left panel), at the onset of acute eosinophilic pneumonia (AEP) (middle panel), and 7‐days after treatment with prednisolone (post‐prednisolone; right panel). Chest radiograph at the onset of AEP shows consolidation in the right upper lobe (Arrow). C and D, Chest computed tomography (CT) after two cycles of nivolumab (left panels), at the onset of AEP (middle panels), and 1‐month after treatment with prednisolone (post‐prednisolone; right panels). Consolidation in right upper lobe (C) and ground‐glass opacity in left lower lobe at the onset of AEP (D) are shown. Arrows indicate consolidation (C) and ground‐glass opacity (D)

A 62‐year‐old man was diagnosed with lung adenocarcinoma and had right lower lobectomy (pT2bN2M0 stage III A, PD‐L1 tumor proportion score <1%; Figure 1A). Thereafter he received postoperative adjuvant therapy of cisplatin and vinorelbine. After 5 months, he was diagnosed with postoperative recurrence of lung adenocarcinoma with multiple metastasis in both lungs. He received platinum‐based chemotherapy as the first‐line chemotherapy regimen and nivolumab was selected as the third‐line regimen. The patient had no history of asthma, atopy, and drug allergy. The patient had not begun taking any new medications and had no history of cigarette smoking.

After three cycles of nivolumab treatment (3 mg/kg every 2 weeks), he presented with cough and chest computed tomography revealed pulmonary infiltrates in both lungs (Figure 1B–D). Levofloxacin was administered for 12 days; however, antibiotics did not improve his symptom or radiological findings. Thus, bronchoalveolar lavage was performed from the right upper lobe. Bronchoalveolar lavage cellular analysis showed a significant increase of total cell count of 12.1 × 10^5^ mL, of which 27.1% were eosinophils (normal upper limit, 1.3%), 8.3% were lymphocytes (normal upper limit, 11%). No pathogenic bacterial organism was cultured. The patient was diagnosed with AEP based on the diagnostic criteria for AEP.^7^ Nivolumab was suspended and the patient was started on oral prednisolone (0.5 mg/kg/day, 30 mg/day), which is tapered 5 mg weekly. His symptoms and radiological findings had rapidly improved (Figure 1B–D), which is consistent with AEP.^5^, ^6^, ^7^ No sign of lung cancer worsening has been observed since the initiation of treatment with nivolumab or prednisolone.

## 3 DISCUSSION

The pathophysiology underlying irAEs has not been fully elucidated.^8^, ^9^, ^10^ Elucidating mechanisms of irAEs is needed to develop precise treatments for irAEs.^8^ PD‐1 and the ligands, PD‐L1 and PD‐L2 have been implicated in playing an important role in maintaining immune homeostasis not only in cancer but also in lung allergic diseases.^2^, ^3^ Th2 cells predominantly produce interleukin‐4 (IL), IL‐5, and IL‐13, which have essential roles in the development of Th2 associated lung diseases, enhancing the growth, differentiation, and eosinophil recruitment.^1^ Recent studies have shown that PD‐L2 is highly expressed on pulmonary dendritic cells (DCs) and inhibits responses of Th2 cells, which express PD‐1, suggesting that blocking the PD‐1–PD‐L2 interaction by anti‐PD‐1 antibodies could facilitate a Th2 inflammation.^1^, ^2^, ^3^, ^11^, ^12^

**Figure 2:**
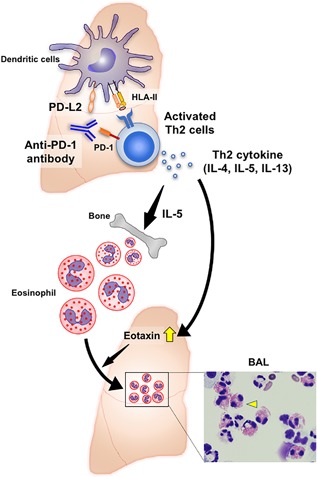
A hypothetical mechanism of the onset of AEP as an irAE in an advanced non‐small cell lung cancer patient based on the current case report and references. Antibodies against PD‐1 inhibit binding of not only PD‐L1 but also PD‐L2 to PD‐1. PD‐L2 is highly expressed on pulmonary DCs and inhibits responses of Th2 cells, which express PD‐1. The blocking the PD‐1–PD‐L2 interaction facilitates a pulmonary Th2‐type inflammation. Th2 cells predominantly produce IL‐4, IL‐5, and IL‐13, which have essential roles in the development of Th2‐associated lung diseases, enhancing the growth, differentiation, and recruitment of eosinophils. One of the Th2 cytokines, IL‐5, supports the development of eosinophils in the bone marrow. Eosinophilia in lung tissue is driven by the recruitment of eosinophils to the lung mucosa and interstitium via production of eotaxin induced by Th2 cytokines in the lung, which results in the onset of AEP as an irAE in lung cancer patients treated with an anti‐PD‐1 antibody therapy. Right lower imaging indicates a photomicrograph of representative bronchoalveolar lavage cytospin preparation, which shows eosinophilia observed in the current case. Yellow arrowhead indicates an eosinophil

Antibodies against PD‐1 inhibit binding of both PD‐L1 and PD‐L2 to PD‐1 expressed on activated T cells.^3^, ^8^, ^12^ This raises the possibility that blockade of PD‐1–PD‐L2 interaction by anti‐PD‐1 antibodies mediates Th2‐associated pulmonary diseases such as AEP: anti‐PD‐1 antibody activates Th2 cells and promotes the production of Th2‐type cytokines in the lung. One of the Th2 cytokines, IL‐5, supports the development of eosinophils in the bone marrow. Eosinophilia in lung tissue is driven by the recruitment of eosinophils to the lung mucosa and interstitium via production of eotaxin induced by Th2 cytokines, which could result in the onset of AEP as an irAE (Figure 2).

## 4 CONCLUSION

In conclusion, the present study describes the first case of AEP as an irAE in a lung cancer patient who had received PD‐1 blockade therapy. The present study also proposes a potential mechanism of the onset of AEP as an irAE. Given the increasing frequency of the use of anti‐PD‐1 antibodies, clinicians should be aware of the risk of AEP as a potential irAE. This study may improve our understanding of the pathophysiology underlying Th2‐associated irAEs and AEP.

## CONFLICT OF INTEREST

No potential conflicts of interest were disclosed.

## ETHICAL STATEMENT

This study was approved by the patient and we obtained written consent form the patient. This report was prepared in accordance with the Helsinki Declaration.

## References

[1] Lambrecht BN , Hammad H . The immunology of asthma. Nat Immunol. 2015; 16:45–56. 25521684 10.1038/ni.3049

[2] Bratke K , Fritz L , Nokodian F , et al. Differential regulation of PD‐1 and its ligands in allergic asthma. Clin Exp Allergy. 2017; 47:1417–1425. 28865147 10.1111/cea.13017

[3] Chen DS , Irving BA , Hodi FS . Molecular pathways: next‐generation immunotherapy‐inhibiting programmed death‐ligand 1 and programmed death‐1. Clin Cancer Res. 2012; 18:6580–6587. 23087408 10.1158/1078-0432.CCR-12-1362

[4] Mato N , Bando M , Kusano A , et al. Clinical significance of interleukin 33 (IL‐33) in patients with eosinophilic pneumonia. Allergol Int. 2013; 62:45–52. 23000728 10.2332/allergolint.12-OA-0439

[5] Allen JN , Pacht ER , Gadek JE , Davis WB . Acute eosinophilic pneumonia as a reversible cause of noninfectious respiratory failure. N Engl J Med. 1989; 321:569–574. 2761601 10.1056/NEJM198908313210903

[6] Rhee CK , Min KH , Yim NY , et al. Clinical characteristics and corticosteroid treatment of acute eosinophilic pneumonia. Eur Respir J. 2013; 41:402–409. 22599359 10.1183/09031936.00221811

[7] De Giacomi F , Decker PA , Vassallo R , Ryu JH . Acute eosinophilic pneumonia: correlation of clinical characteristics with underlying cause. Chest 2017; 152:379–385. 28286263 10.1016/j.chest.2017.03.001

[8] Postow MA , Sidlow R , Hellmann MD . Immune‐related adverse events associated with immune checkpoint blockade. N Engl J Med. 2018; 378:158–168. 29320654 10.1056/NEJMra1703481

[9] Tomita Y , Sueta D , Kakiuchi Y , et al. Acute coronary syndrome as a possible immune‐related adverse event in a lung cancer patient achieving a complete response to anti‐PD‐1 immune checkpoint antibody. Ann Oncol. 2017; 28:2893–2895. 28651328 10.1093/annonc/mdx326

[10] Horio Y , Takamatsu K , Tamanoi D , et al. Trousseau's syndrome triggered by an immune checkpoint blockade in a non‐small cell lung cancer patient. Eur J Immunol. 2018; 48:1764–1767. 29981156 10.1002/eji.201847645

[11] van der Werf N , Redpath S A , Azuma M , Yagita H , Taylor MD . Th2 cell‐intrinsic hypo‐responsiveness determines susceptibility to helminth infection. PLoS Pathog. 2013; 9:e1003215. 23516361 10.1371/journal.ppat.1003215 PMC3597521

[12] Zhang Y , Chung Y , Bishop C , et al. Regulation of T cell activation and tolerance by PDL2. Proc Natl Acad Sci USA. 2006; 103:11695–11700. 16864790 10.1073/pnas.0601347103 PMC1544232

